# Cyclooxygenase and Neuroinflammation in Parkinson’s Disease Neurodegeneration

**DOI:** 10.2174/157015910790909485

**Published:** 2010-03

**Authors:** Anna L Bartels, Klaus L Leenders

**Affiliations:** Dept. of Neurology, University Medical Centre Groningen, Hanzeplein 1, 9700 RB Groningen, The Netherlands

**Keywords:** COX-2, Parkinson, neuroinflammation, microglia, neurodegeneration, neuroprotection.

## Abstract

Cyclooxygenase (COX) expression in the brain is associated with pro-inflammatory activities, which are instrumental in neurodegenerative processes such as Parkinson’s disease (PD). It is discussed that drugs with the capacity to rescue dopaminergic neurons from microglia toxicity and neuroinflammatory processes may result in an amelioration of parkinsonian symptoms by delaying the onset or slowing progression. This article reviews the involvement of COX in neuroinflammation, specifically focussing at the role of selective COX-2 inhibition in neuroinflammation and neurodegeneration in Parkinson’s disease.

## INTRODUCTION

Parkinson’s disease (PD) is one of the most common neurodegenerative diseases, and is characterised by the progressive loss of dopaminergic and other catecholaminergic neurons and projections from brain stem regions [11]. Research on the aetiology of PD has resulted in an abundance of information on neurodegenerative processes, but still little is known about the events causing the initiation and also the progression of the disease. PD neurodegeneration was previously considered to be a purely neuronal process, but is now seen as the result of multiple pathogenic factors. In recent years, the cross talk between neurons and glia has become an intensive research focus for the understanding of brain pathophysiology. More specifically, neuroinflammatory processes involving an increased expression of cyclooxygenase (COX) and elevated prostaglandin E2 (PGE2) levels have been associated with several neurodegenerative diseases, such as PD, Alzheimer’s disease (AD) and amyotrophic lateral sclerosis (ALS) [39].

COX comes in two isoforms: COX-1, which is widely distributed in virtually all cell types and is thought to mediate physiological responses, and COX-2, an inducible form. COX-2 is rapidly expressed in several cell types in response to cytokines, growth factors and pro-inflammatory molecules. COX-2 has emerged as the isoform primarily responsible for prostanoid production in acute and chronic inflammatory conditions. Thus, COX-2 may contribute to neurodegenerative processes. However, in spite of intense research in the last decade, the evidence for a direct role of COX-2 in neurodegenerative disease and neuroprotective effect of COX-2 inhibition is still controversial. This article will review the role of COX-2 in neurodegenerative diseases with the main focus on its possible roles in PD progression.

## COX IN PD: EPIDEMIOLOGICAL STUDIES

The hypothesis that neuroinflammation is a key component in the progression of Parkinson’s disease, has been corroborated by several epidemiological studies. To date, nine epidemiological studies have investigated the association between regular NSAID use and risk of PD. The conventional NSAID ibuprofen has the strongest epidemiological support for risk reduction of PD development [16, 17]. In the first study, more than 44000 men and nearly 99000 women were followed for 14 and 18 years, respectively. A total of 236 men and 179 women developed PD. The risk of developing PD was 45 % lower among regular users of non-aspirin NSAIDs and in those who took two or more tablets of aspirin a day [17]. In a subsequent study with a cohort of 146565 people, ibuprofen was associated with 35 % lower risk of PD. There was a significant trend for lower risk with increasing consumption of ibuprofen (from relative risk (RR) 0.73 with fewer than 2 tablets per week to RR 0.61 for daily use) [16]. No associations were found for aspirin or other NSAIDs, although it should be noted that most people in the cohort used ibuprofen. More recently, a population-based study in 293 PD cases and 286 controls showed protective effect for aspirin users only in women (OR 0,51) and a stronger effect for non-aspirin NSAID users without gender difference, particularly for those who reported more than two years of use (OR 0,44) [59].

However, in other studies findings were controversial. A case-control study in 1258 PD patients and 6638 controls reported that non-aspirin NSAID use reduced PD risk only in men but surprisingly did the opposite in women. Use of NSAIDs was associated with a 20 % reduction of PD development in men and a 20 % increase in women [29]. A smaller study in 392 subjects found that PD cases less frequently used NSAIDs than controls, however this difference did not reach significance. They also showed a significant association between pre-existing immune-related diseases and development of PD, especially in women [10], which may support the hypothesis of an inflammatory component in the pathogenesis of PD. Another case-control study in 206 PD patients and 383 controls, however, provided limited support for the hypothesis of PD risk reduction by aspirin, and no indication of protection by other NSAIDs [55]. A family-based case control study in 356 PD patients and 317 family controls revealed inverse associations of smoking and caffeine consumption with PD, but not of NSAID use [28]. Another group that conducted a nested case-control study using an anti-hypertensive agent drug database found no protective effect of past NSAID users and even a slightly higher risk of developing PD in current NSAID users (RR 1,49) [25]. Recently, in a large cohort study assessing 697.078 subjects these authors did not find decreased risk for PD development with NSAID use [24]. Limitations of these studies were the inability to assess the use of over-the-counter NSAIDs and inability to adjust for other potential PD risk factors as confounders.

Notably, the subjects in these studies used non-selective COX inhibitors. In the study of Chen *et al*, the non-selective COX inhibitor ibuprofen showed the strongest effect in comparison with the COX-1 inhibitor aspirin. However, most subjects in that study used ibuprofen. Selective COX-2 inhibitors have only recently been introduced and are mostly used as second choice after the traditional non-selective NSAIDs. Until now, no study has been performed to investigate the influence of selective COX-2 inhibition on PD incidence or PD progression.

## COX-1 AND COX-2 IN THE BRAIN

COX, also known as prostaglandin (PG) H synthase, catalyses the first step in the synthesis of prostanoids from arachidonic acid (AA). COX exhibits two catalytic activities: a bis-oxygenase activity (cyclooxygenase), which catalyses PGG2 formation from AA, and a peroxidase activity, which reduces PGG2 to PGH2. The peroxidase activity also results in the production of free radicals. Through the intermediate of PGH2, COX enzymes produce 5 prostanoids: PGE2, PGF2, PGD2, PGI2 (prostacyclin) and thromboxane A2 (TxA2). PGs play a pivotal role in the biochemical mechanisms that induce pain, hyperpyrexia, inflammatory cytoprotective and cytotoxic processes. Finally, during the cyclooxygenase activity, COX undergoes a conformational rearrangement leading to an unstable intermediate. This process is called “suicide inactivation” and it limits prostanoids synthesis [49].

In the majority of tissues, COX-1 appears to be the only isoform constitutively expressed, confirming its role in physiological functions through homeostatic PG synthesis. However, in brain, testes and kidney cells, both COX-1 and COX-2 were found to be expressed under physiological conditions [49]. In rat brain, COX-1 and COX-2 expression is present in distinct areas of cerebral cortex and in hippocampus. In the midbrain, pons and medulla, COX-1 prevails [12]. Also in several regions of the human brain both COX-1 and COX-2 expression are present, with COX-2 expression being most prominent in the hippocampus [63]. Recently, also a third variant of COX, named COX-3 has been identified [15]. COX-3 is a product of the COX-1 gene and has the expression in the brain, mainly in the cerebral cortex. As COX-1, COX-3 is not induced by acute inflammatory stimulation [46]. Enzymatic activity of COX-3 is glycosylation-dependent and especially sensitive to the inhibitory activity of acetaminophen (paracetamol) [15]. COX-3 may represent the brain-specific COX isoform, explaining the potent analgetic and antipyretic actions of paracetamol in spite of its poor ability to inhibit COX in peripheral tissues. However, its functional role in the brain is largely unknown.

The potential role of COX isoforms in brain pathological conditions has been extensively reviewed in the past years. Only COX-2 is dramatically up-regulated during inflammatory processes, which led to the concept that selective COX-2 inhibition can reduce inflammation without affecting the physiological functions of COX-1. On the other hand, it has recently been brought forward that COX-1 could be the mayor player in neuroinflammation by being predominantly localised in microglia and thus secrete PGs in response to microglia activation. COX-2, which is mainly localised in neurons, is expected to increase PG synthesis in response to neuronal insults [18]. Consistent with a suggested role of COX-2 in excitotoxic neuronal injury, COX-2 is expressed in the postsynaptic neuronal cell bodies of glutamatergic synapses [34].

Potential harmful downstream effectors of COX-2 toxicity are PGE2 and free radicals. PGE2 has an enhancing effect on glutamate release, leading to neurotoxic levels of glutamate. COX-2 could also contribute to oxidative stress-mediated damage by producing oxidizing reactive species during the peroxidase activity. However, the beneficial or detrimental role of COX-2 in inflammatory and neurodegenerative processes is still controversial. Several studies suggest a special role for COX-2 in normal neural synaptic function. The “constitutive” cerebral COX-2 expression is dependent on normal synaptic activity and is dynamically regulated with rapid increases during seizures or ischemia, while down-regulation occurred by glucocorticoids [62]. COX-2 immunoreactivity is seen in distal dentrites and dendritic spines, localized to excitatory glutamatergic neurons, supporting the role of COX-2 in synaptic activity. Furthermore, COX-2 inhibition with celecoxib impaired spatial memory in rats in an experiment using the Morris water maze, a hippocampal-dependent learning task [52]. Also ibuprofen, a non-selective COX-inhibitor, caused memory consolidation deficits in this experimental setting, which was associated with decreased expression of brain-derived growth factor (BDNF) [47]. COX-2 derived PGE2 could participate in synaptic plasticity through modulation of adrenergic, noradrenergic and glutamatergic neurotransmission and regulation of membrane excitability. Moreover, PGE2 is involved in the coupling of synaptic plasticity with cerebral blood flow, resulting in a hyperaemic response [42]. 

In experimental models with neuronal insults, such as ischemia, COX-2 inhibition provides protection from neuronal damage in the early phase of damage, without attenuating inflammatory gene expression [36]. In contrast, in experimental models of primary neuroinflammation, such as lipopolysaccharide (LPS), detrimental effect of COX-2 inhibition is seen with increased glial activation and inflammatory markers [1]. This could be due to anti-inflammatory properties of COX-2, which have been coupled to the production of specific prostaglandin metabolites by COX-2, the cyclopentanone prostaglandins [61], which are ligands of the peroxisome proliferator-activated receptor-γ (PPAR-γ). However, in neurons, cyclopentanone prostaglandins were reported to induce apoptosis [44]. Thus, in neurodegenerative processes COX-2 could play a role in triggering neural cell death *via *its production of cyclopentanone prostaglandins.

In clinical trials that assessed long-term safety, selective COX-2 inhibitors were associated with cardiovascular adverse events. The VIGOR study reported a higher incidence of myocardial infarction in arthritis patients using rofecoxib compared to naproxen (0,4 vs 0,1 %) [7]. Retrospective analysis revealed that 35 % of these infarctions occurred in the 4 % of patients who in retrospect had been candidates for low-dose aspirin. It should also be noted that cardiovascular morbidity is increased in autoimmune diseases, especially rheumatoid arthritis. In the CLASS study, the incidence of cardiovascular events in the celecoxib group was equal to that in the classic NSAID group [48]. A meta-analysis of clinical literature on COX-2 inhibitors concluded that celecoxib is the safest COX-2 inhibitor relating to the cardiovascular safety data [33].

Thus, potential differential effects of COX-2 inhibition in neurodegenerative processes need to be considered to differentiate neuroprotective mechanisms from possibly harmful effects. Below, we will specifically focus on the evidence of COX-2 involvement in experimental PD models.

## COX-2 IN PARKINSON’S DISEASE

Human and animal studies have shown robust microglia activation in PD, suggesting an important role of these cells in the pathogenesis of PD. On the contrary, astrogliosis has been sporadically observed [41]. Interaction of microglial cells with apoptotic neurons has been reported to selectively promote COX-2 expression and PGE2 synthesis [20]. COX-2 may mediate microglial activation and may play a key role in amplifying the inflammatory response with toxic effects. COX-2 may contribute to the progression of neurodegeneration through the production of toxic free radicals and increasing local glutamate concentration to toxic levels [6]. 

Interestingly, the healthy substantia nigra (SN) exhibits the highest concentration of microglia in the brain, which increases with age [5]. Besides being prone to deleterious effects from oxidant stress by the high glutathione and iron concentrations in dopaminergic neurons [32], the SN is thus also sensitive to neuroinflammation. In post-mortem PD brains, the SN showed a high amount of activated microglia [38], suggesting involvement of these cells in the neurodegenerative process. Activated microglia are recruited to the SN and stuck to dopaminergic neurons. Once activated, glial cells become phagocytes that ingest degenerating neurons. This process occurs early in neurodegeneration and starts at the extending fibres, such as the dendrites [51]. Hence, detrimental compounds are released in this process, such as interleukin (IL)-1β, IL-6, tumor necrosis factor-α (TNF-α) and interferon-γ (IFN-γ), which may stimulate inducible nitric oxide synthase (iNOS) or activate receptors involved in apoptosis [54]. Another pathway that links inflammation and cell death involves NF-κB, which is known for its role in preventing apoptotic cell death. In a chronic MPTP model of PD, activation of NF-κB was revealed [22]. The synthesis of COX-2 is promoted by NF-κB as well as pro-inflammatory cytokines such as TNF-α, or *via *the c-Jun N-terminal kinase (JNK) pathway [53].

Increased susceptibility to excitotoxicity in COX-2 overexpressing neurons and neuroprotection by COX-2 inhibition has been shown in several experimental PD models [39]. Furthermore, the specific involvement of COX-2 in PD neurodegeneration is supported by the observation that MPTP neurodegeneration was mitigated in COX-2 knock out, but not in COX-1 knock out mice [26]. 

Increased microglial COX-2 expression was reported in post-mortem SN from 11 PD patients, whereas neuronal and astroglial COX-2 expression did not differ between PD patients and control subjects. It was also seen that moderate COX-1 reactivity in neuronal somata and in few glial cells was similar in PD and control subjects [35]. However, another study showed COX-2 to be specifically induced in dopaminergic neurons in the SN in post-mortem PD subjects and in the 1-methyl-4-phenyl-1,2,3,6-tetrahydropyridine (MPTP) mouse model, whereas no obvious staining of astrocytes or activated microglia was detected [53]. On the other hand, in a 6-hydroxydopamine (6-OHDA) PD model study, celecoxib was shown to mitigate 6-OHDA induced microglial activation [45]. A recent study claims that the COX-2 specific NSAID valdecoxib also significantly mitigated microglial activation in a MPTP mouse model [57]. 

An explanation for the different findings of neuroprotective effect in several studies of neuroinflammation and neurodegeneration may be in the timing of treatment. In most toxic PD model studies, anti-inflammatory treatment is started before or at the time of toxic lesion, which may be important to diminish early damaging reactions. This idea is supported by a study of Shriram *et al.*, showing that minocycline treatment after MPTP lesion attenuated microglial activation, but failed to afford neuroprotection [50]. Their findings suggest that attenuation of microglial activation may be insufficient to modulate neurotoxicity when transient activation of microglia may suffice to initiate neurodegeneration. 

On the other hand, microglia independent neuroprotective effects of COX-2 inhibition have been reported. Celecoxib was recently shown to have an attenuating effect on LPS induced nigrostriatal neurodegeneration without affecting microglial activation [30]. Also other studies suggest that the effect of COX-2 modulation may be independent from microglia activity. A study with 6-OHDA exposed neuronal cultures showed 2-fold higher prostaglandin (PG) levels, and prevention of PG increase by ibuprofen was inversely correlated to dopaminergic cell loss. Thus the rise in PG levels with 6-OHDA exposure was not due to microglial activation in this *in vitro* model [13]. Wang *et al.*, [60] however showed that MPTP increased PG in mixed neuron-microglia cultures, but not in neuron -, microglia -, or astroglia alone cultures, pointing at the importance of interaction between neurons and glial cells. PG increase was abolished by treatment with DuP697, a COX-2 selective inhibitor, which also reduced dopaminergic neurotoxicity [60]. Microglia-independent effect of COX modulation on neuronal cell death has also been suggested in an *in vivo* MPTP model. The selective COX-2 inhibitor rofecoxib blocked ventral midbrain PG production in MPTP injected mice and attenuated neuron and fiber loss, demonstrating the crucial enzymatic function of COX-2 to its neurotoxic effects on dopaminergic neurons. This report suggested that the neuroprotective effect of COX-2 inhibition was related to the blockade of COX-2-mediated dopamine oxidation and not to decreased microglial activation [53]. Moreover, a recent study showed that COX-2 facilitated dopamine oxidation in a cell-free system and in COX-2 overexpressing SH-SY5Y cells, which was blocked by the COX-2 inhibitor meloxicam. This was accompanied by accumulation of α-synuclein oligomers, which is an early step in PD pathogenesis [14]. These findings suggest that increased COX-2 in dopaminergic cells under stressful conditions can facilitate dopamine oxidation to quinone species, triggering oxidative stress, and that COX-2 overexpression in dopaminergic cells may also play a role in α-synuclein accumulation.

In addition to these studies, we have recently performed a study with celecoxib treatment started after 6-OHDA striatal lesion in rats, and could not confirm a diminishing effect on microglia activation (submitted data). The aim of that study was to diminish neuroinflammation by COX-2 inhibition, and thereby improve blood-brain barrier (BBB) P-glycoprotein (P-gp) efflux function. However, this study showed decreased P-gp up-regulation after COX-2 inhibition by celecoxib. BBB P-gp efflux function is thought to play a role in the detoxification of the brain in several neurodegenerative diseases [3, 58]. This adverse effect of COX-2 inhibition, which is also described in epilepsy models [4], may hamper the neuroprotective potential of COX-2 inhibition in neurodegenerative disease. Another *in vivo* study in rats, however, did not show a substantial effect of celecoxib on P-gp mediated efflux [21]. The discrepant findings relating to microglia involvement remain to be reconciled; however, several studies indicate that microglia-independent actions of COX-2 could play key roles in PD neurodegeneration (see also Fig. (**1**). Microglia and COX involvement in neuronal injury). 

It should also be borne in mind that interactions between apoptotic neurons and microglia can also lead microglia to acquire an anti-inflammatory phenotype with potential neuroprotective properties. Minghetti’s group has provided evidence that under chronic stimulation a progressive down-regulation of glial pro-inflammatory molecule expression is seen, while the synthesis of other products with potential protective activities is stimulated [40]. Furthermore, relating to COX-2 activity, it has been found that genetic deletion of the PGE2 receptor exacerbated neuronal damage, suggesting a potential protective effect of PGE2 receptor activity [37]. Finally, it has been demonstrated that COX-2 induction in inflammation is expressed chronically and is also observed during the resolution of inflammation and during healing of wounds [19]. In chronic inflammation, COX-2 inhibition may lead to exacerbation of inflammation [1]. Thus, it is important to determine whether COX-2 inhibition in a chronic neuroinflammatory process will attenuate inflammation or might have differential effects.

## CONCLUDING REMARKS

When these experimental findings have to be translated to the human disease, questions that remain are whether COX-2 inhibition could be effective to decrease PD risk and progression, or whether indeed undesired effects can be seen relating to inhibition of inflammatory processes in neurodegeneration. It needs to be further investigated whether microglia activation is involved in disease progression in PD patients. *In vivo* PET imaging studies have shown increased microglia activation in PD patients; however its relation to disease progression is still unclear [27, 43]. Furthermore, these methods are so far insufficient to quantify microglia activation and measure a possible effect of anti-inflammatory treatment in patients [2]. New PET tracers for quantification of microglia activation are being developed for use in patients [8, 9, 23, 31, 56] and may help to further elucidate these issues in the human disease situation. On the other hand, COX-1 is predominantly localized in microglia and could be a mayor player in the glial inflammatory response. The epidemiological evidence may point at a decreased risk of PD development by non-selective NSAID use rather than decreased progression of the disease. However, the potential protective effect of COX-1 inhibition as well as COX-independent effects of several NSAIDs in PD needs to be further investigated. 

Local inflammatory reactions of the human brain may persist for much longer than in experimental rodent models. As a consequence, this may lengthen the release of toxic substances subsequent to the acute inflammatory response. It is hypothesised that a harmful substance first induces reactive microgliosis and secretion of its pro-inflammatory factors, such as PGE2 and cytokines. These may enhance COX-2 dopaminergic neuronal activity and lead to a progressive wave of neuronal damage, and so on. As COX-1 is mainly expressed on microglia, early damage of an insult to the SN may be diminished mainly by COX-1 inhibition to decrease reactive microgliosis. COX-2 inhibition is hypothesised to diminish neuronal damage through microglia-independent mechanisms such as COX-2 mediated dopamine oxidation. However, the potential of exacerbation of a chronic inflammatory reaction by COX-2 inhibition in the human disease situation warrants further research, which could be aided by *in vivo* PET imaging in rodent models as well as patients. Furthermore, the possible side effect of COX-2 inhibitors of inhibiting brain protective P-gp function may devaluate its neuroprotective potential in neurodegenerative disease. So far, the discrepant findings relating to the effect of COX-2 inhibition preclude the set-up of clinical trials in PD and warrant further investigation of the roles of the COX isomers in neuroinflammatory and neurodegenerative processes.

## Figures and Tables

**Fig. (1):**
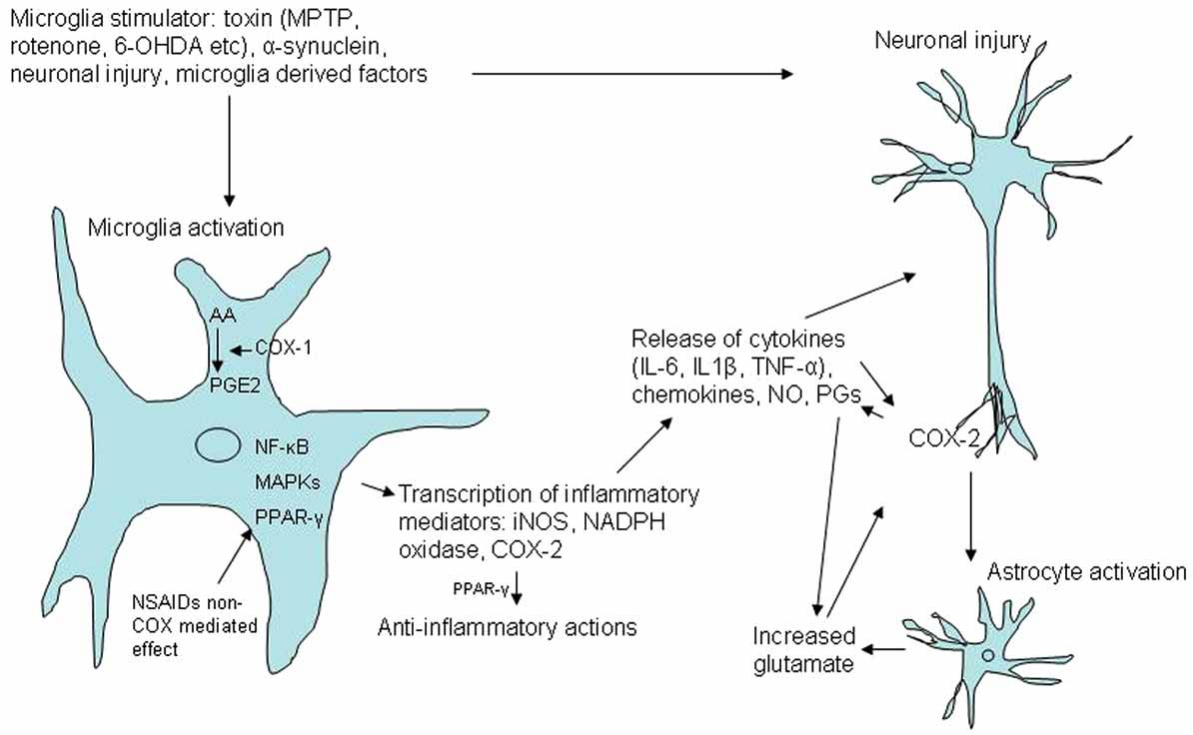
Microglia and COX involvement in neuronal injury. Various stimuli can activate microglia in Parkinson’s disease, including aggregated α-synuclein, toxins (such as MPTP, 6-OHDA, LPS, rotenone, paraquat, pesticides). Intracellular signalling cascades involving NF-κB and MAP kinases lead to microglial activation and induction of proinflammatory mediators, including iNOS, NADPH oxidase and COX-2, and the subsequent release of cytokines (e.g. IL-1β, IL-6, TNF-α), nitric oxide (NO) and prostaglandins (including PGE2). COX-1 is constitutively expressed on microglia and may be the primary source of PGE2 release in early inflammation phases. COX-2 is localized in neurons and contributes to PGE synthesis in response to neuronal insults. Astrocyte activation may increase glutamate levels with cytokine and COX-2 induction.
